# Prevalence of Vitamin D Deficiency among Hemodialysis Patients in Palestine: A Cross-Sectional Study

**DOI:** 10.1155/2021/6684276

**Published:** 2021-03-12

**Authors:** Zaher A. Nazzal, Zakaria Hamdan, Nihal Natour, Maram Barbar, Rawan Rimawi, Eziyeh Salaymeh

**Affiliations:** ^1^Department of Medicine, Faculty of Medicine and Health Sciences, An-Najah National University, Nablus 44839, State of Palestine; ^2^Department of Nephrology, An-Najah National University Hospital, Nablus 44839, State of Palestine

## Abstract

**Introduction:**

The level of vitamin D status and its relationship to kidney function and liver function among patients with and without type 2 diabetes were not studied among Palestinian hemodialysis patients before. The aim of this study was to assess the status of vitamin D in hemodialysis patients with and without type 2 diabetes and its determinants.

**Methods:**

Data were collected on 163 patients on hemodialysis therapy in the Nephrology Department at Najah National University Hospital. Information on age, sex, plasma 25 (OH)D, serum calcium, serum phosphate, parathyroid hormone, dialysis period, hypertension, diabetes, ALT, AST, albumin, alkaline phosphates, and BMI was obtained from the medical records. Data were analyzed using SPSS. *Findings*. The mean level of 25 (OH)D was 17.3 ± 10.5 ng/ml. Only 12.9% of subjects had 25 (OH)D levels >30 ng/ml, whereas 65% had levels between 10 and 30 ng/ml; the remaining 22.1% were severely vitamin D deficient (<10 ng/ml). Vitamin D deficiency was more prevalent among females. It was not related to PTH, calcium, kidney, or liver function tests.

**Conclusion:**

Vitamin D deficiency is highly prevalent among patients on hemodialysis with or without DM2.

## 1. Introduction

Vitamin D is a hormonal vitamin that exerts its function through endocrine [1] and paracrine manner [2]. In nucleus, vitamin D performs its function through vitamin D receptors that modify many genes' transcription. Vitamin D's primary role is to change calcium absorption and metabolism with a net increase in bone mineral density, the classical function of vitamin D [3]. However, it has many other roles, including its novel function in insulin resistance and secretion, with some studies indicating a protective role for vitamin D from diabetes mellitus (DM) [4].

Renal and extrarenal enzymatic pathways exist for the activation of vitamin D and calcitriol production, which is the most active form of vitamin D [4]. Calcitriol and parathyroid hormone (PTH), among others, are associated with tight regulation of ionized calcium. Optimal levels of both 25-hydroxyvitamin D and calcitriol are associated with improved calcium and phosphorus homeostasis [5].

Patients on hemodialysis usually have low vitamin D status [6] and higher PTH [7]. Metabolic disturbances in vitamin D status and PTH in hemodialysis patients are associated with increased mortality and decrease in the quality of life [8], and hence the use of supplementation among dialysis patients is common. Despite 1,25-vitamin D supplements, many patients with ESRD on dialysis will develop reduced bone mineral density [9]. Vitamin D supplementation in the form of calcitriol and paricalcitol improves patients' survival on hemodialysis [10]. Patients on hemodialysis in NHANES III have higher mortality from cardiovascular disease associated with vitamin D deficiency [11], which is linked to vitamin D underlying conditions such as hypertension, insulin resistance, diabetes, and dyslipidemia [12, 13].

Diabetes mellitus (DM2) is the seventh cause of mortality among the Palestinian population, and according to the Palestinian bureau of statistics, 9.1% of the Palestinian people who are between the ages of 20–79 years have DM2 [14]. DM2 has many complications, including nephropathy, which happened at a rate as high as 34.6% among patients with DM2 in Palestine [15] with the risk of developing end-stage renal disease and consequent use of hemodialysis, for which there is a prevalence of 240.3 per million population [16] in the Palestine.

Nablus is part of the Occupied Palestinian Territories (OPT), where it is expected that low vitamin D status is a widely prevalent health problem despite ample sunshine. In OPT, fortification of food items such as a dairy product with vitamin D is not a published policy. According to the author knowledge, some marketed dairy products that may not be well purchased by the public may be supplemented with vitamin D. Hence, the goals of the study are to (1) provide preliminary data on vitamin D status and PTH levels among a group of Palestinian patients on hemodialysis with and without DM2, (2) study the association between vitamin D status and indicators of calcium homeostasis, and liver function, and (3) compare vitamin D status levels between patients with and without DM2.

## 2. Materials and Methods

In a cross-sectional study design, we evaluated the charts of 163 patients with measured plasma 25-hydroxy vitamin D (25 (OH)D) as it is the best method for defining a person's vitamin D status (16). The study was conducted at the Hemodialysis Unit in the Department of Nephrology at the Najah National University Hospital (NNUH). Its dialysis unit is one of the largest hemodialysis units in the West Bank, with a capacity of 332 dialysis patients.

Information on age, sex, plasma 25 (OH)D, serum calcium (8.0–10.0 mg/dL) (17), serum phosphate (3–4.5 mg/dL) (18), parathyroid hormone (10–65 mg/dL) (19), dialysis period, hypertension, DM, alanine transaminase (ALT), aspartate transaminase (AST), albumin, alkaline phosphates, and BMI was obtained from the medical records. BMI <30 was considered non-obese, and BMI ≥30 was considered obese (21). Based on clinical definitions and for purposes of interpretability, 25D levels ≥30 ng/dL were considered replete, whereas vitamin D deficiency was defined as level <30 ng/dL and severe deficiency as levels <10 ng/dL (12). Vitamin D levels and other minerals were measured through a blood test.

Our sample was a convenience sample of 163 hemodialysis patients. The patients who did not want to participate in the research, those on 25 (OH) Vitamin D supplements, and patients with chronic liver failure were excluded. Approval from An-Najah National University Institutional Review Board (IRB) was taken. Permission from NNUH to access the data we need was obtained; no consent was taken because we did the test on a blood sample that is routinely drawn from patients.

SPSS V.20 was used to analyze data. Normally distributed variables were expressed as mean ± standard deviation, and non-normally distributed variables were expressed as median and range (minimum and maximum). Categorical data were described as numbers and percentages. *P* values < 0.05 were considered statistically significant. The Kruskal–Wallis test or Chi-square tests were used to examine variation between the three groups. Since several values are not normally distributed, Spearman's rank correlation (*r*) has been used to establish the univariate correlations between 25 (OH)D and selected parameters. Linear regression analysis was conducted to assess the relationship between 25 (OH)D and clinical parameter including age, sex, DM, years on dialysis, calcium, phosphorous, ALP, serum albumin, and serum PTH. Variables included in the model were selected based on previously identified predictors in the literature.

## 3. Results

### 3.1. Patients' Characteristics

The sample included 163 consecutive patients from NNUH hemodialysis centers. Their baseline characteristics are presented in Table 1. Mean age of patients was 57.8 ± 15.4, and 62.6% of patients were males. Patients were divided into three groups according to 25 (OH)D levels, a serum 25 (OH)D level of <10 mg/mL was identified as vitamin D severe deficiency, a serum level of ≥10 and ≤30 ng/mL was identified as deficiency, and a serum level of >30 was considered normal.

**Figure 1:**
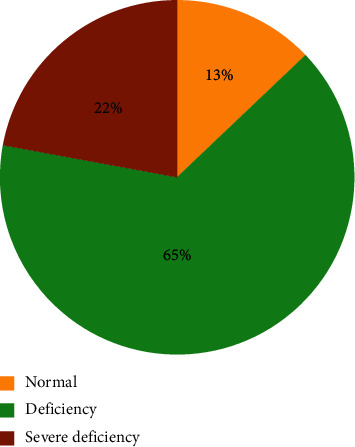
Distribution of 25 (OH)D values among 163 hemodialysis patients.

The mean level of 25 (OH)D was 17.3 ± 10.5 ng/ml. Only 12.9% of subjects had 25 (OH)D levels >30 ng/ml, whereas 65% had levels between 10 and 30 ng/ml; the remaining 22.1% were severely vitamin D deficient (<10 ng/ml) (Figure 1).

### 3.2. Factors Associated with Vitamin D Deficiency

For age, no significant difference was found between the means among the 3 vitamin D groups. Compared with men, women were more likely to be severely 25 (OH)D deficient (14.7 vs 34.4%; *P* < 0.25). Compared to patients without diabetes, those with diabetes were more likely to be severely 25 (OH)D deficient (26.5 vs 38.9%; *P* > 0.05). Also, those with hypertension were more likely to be severely 25 (OH)D deficient (22.4 vs 11.1%; *P* > 0.05). Obese patients were more likely to be severely 25 (OH)D deficient compared to patients with normal BMI (25.4% vs 45.5% *P* > 0.05) (Table 2).

For the biochemical parameters, no significant difference was found between their means among the three vitamin D groups (Table 3).

The Spearman's rank correlation was used to assess the correlation between clinical and biochemical parameters and 25 (OH)D levels. No significant correlation was found with serum levels of ALT (*r* = 0.1), AST (*r* = 0.132), calcium (*r* = 0), phosphorus (*r* = 0.034), parathyroid hormone (*r* = 0.003), ALP (*r* = 0.05), albumin (*r* = 0.04), and dialysis period (*r* = 0.031) (Table 4).

Multiple linear regression model was used to assess predictors of 25 (OH)D levels. Gender was found to be significantly correlated with serum 25 (OH)D level (Table 5).

## 4. Discussion

In a study among Palestinian patients on hemodialysis, vitamin D deficiency and severe deficiency were present modestly. However, vitamin D was not significantly related to PTH and serum calcium, indicating that the deficiency level possibly was associated with concurrent modification of calcium homeostasis. Among the studied groups, kidney and liver function tests were not related to vitamin D in the unadjusted and adjusted model (data are not shown). At the same time, vitamin D was not associated with BMI of the studied group. In this study, vitamin D, PTH, and serum calcium were higher in the group of patients without DM2, which happened to have more prolonged duration dialysis in the same group of patients.

Vitamin D level for patients without DM2 was 18.97 ng/ml, whereas the level for patients with DM2 was 15.7 ng/ml. The high prevalence of vitamin D deficiency and insufficiency was common in our study patients, similar to what was reported among other ethnic groups [17]. In a study performed by Krause et al., patients with end-stage renal disease benefited from supplementation with vitamin D and ultraviolet (UV) radiation of the skin, with UV radiation being seven times more effective than regular supplementation [18]. In a country with ample sunshine like Palestine, located in the Middle East [19], this could be a potential way to enhance vitamin D status and, hence, the quality of dialysis patients' lives [20]. Aside from dialysis patients, reported levels of vitamin D in other groups indicate a severe deficiency in Palestine's patients, which is plausibly related to lack of clear fortification policies and few dietary resources [21].

Our study shows no significant association between PTH and vitamin D status despite high deficiency levels. PTH is known to be elevated in dialysis patients, resulting in a detrimental effect on bone health [22]. Hyperparathyroidism in patients with renal dialysis is commonly treated by active vitamin D [23]. The use of calcitriol supplementation and calcium binder to decrease phosphorus and treat high PTH could cause a lack of association between PTH and vitamin D status. Besides the effect of high PTH on bones, more elevated PTH is linked to CVD [24].

It is well-known that vitamin D's conversion into active calcitriol is impaired in patients with end-stage kidney diseases. This leads to an increase in PTH and a decrease in calcium absorption and reduces phosphorous excretion [25]. Data on calcitriol was not measured in our study. However, most dialysis patients need supplementation of calcitriol, or other forms of active vitamin D. Use of active vitamin D in patients with dialysis is suggested to decrease oxidative stress and inflammation and decrease CVD risk [26].

In a study by Namyr et al. among 516 chronic kidney disease patients, an increment of 10 ng/ml in 25 (OH)D was associated with 25% reduced mortality [27]. In another study, vitamin D supplementation was associated with 38% reduction in all-cause mortality and 45% reduction in CVD mortality [28].

In univariate analysis, there was no difference in vitamin D status among patients with or without DM2. At the same time, dialysis duration was longer in patients without DM2, and GFR was lower. Vitamin D receptor and activating enzymes were expressed in many tissues, including muscles, pancreatic cells, and other tissues implicated in insulin secretion and resistance, which were reviewed elsewhere [4].

Liver enzymes, AST and ALT, are increased in patients who undergo dialysis, as was described earlier [29]. In recent work, elevated AST/ALT ratio was associated with an increase in mortality in hemodialysis patients due to CVD, which could indicate heart tissue injury [30]. Vitamin D was not significantly related to AST/ALT ratio and the significant association with AST and ALT values seem to be not clinically meaningful. ALP was notably high in our study relative to what others reported (64 (52–82 U/L)). High value is linked to high mortality in peritoneal patients, mainly related to CVD [31, 32]. Although our patients had higher ALP values, the AST, ALP, and AST/ALP values matched what others found in dialysis patients [33].

This study is not without limitations, including its cross-sectional design. Intake of calcitriol and calcium binders was not accounted for. In summary, in a group of patients with DM2 and without DM2, who receive dialysis, vitamin D deficiency was very common. Vitamin D status was not correlated with PTH. There was no association between kidney function, liver function, and vitamin D status in univariate analysis.

## Figures and Tables

**Table 1:** Background, clinical, and lab characteristics of study participants (*n* = 163).

Demographic characteristics	
Age	57.8 ± 15.4
Gender (women %)	37.4
BMI	22.2 ± 4.9

Comorbid conditions	
Diabetes (yes %)	50.9
Hypertension (yes %)	87.7
Years on dialysis	4.59 ± 3.5

Laboratory test results	
25 (OH) vitamin D	17.3 + 10.5
Calcium	8.9 ± 0.9
Phosphorus	4.68 ± 1.2
PTH	469.5 ± 563.3
ALP	136.7 ± 211.94
ALT	9.5 (3.0–44.0)
AST	13.52 ± 5.7
AST/ALT	1.32 + 0.54
Albumin	3.73 ± 0.4

Results are expressed as mean ± SD or medians (interquartile ranges) as appropriate, PTH: parathyroid hormone; ALP: alkaline phosphatase; ALT: alanine transaminase; AST: aspartate transaminase.

**Table 2:** Background and co-morbidities of dialysis patients according to serum 25 (OH)D levels.

	Severely deficient <10 ng/ml	Deficient 10–30 ng/ml	Normal >30 ng/ml	*P* values^*∗∗*^
	*n* = 36	*n* = 106	*n* = 21	
Age	59.5 + 13.9	57.5 + 21.2	59.2 + 15.4	0.723∗

Gender				
Female	21 (34.4%)	32 (52.5%)	8 (13.1%)	0.011
Male	15 (14.7%)	74 (72.5%)	13 (12.7%)	

Hypertension				
Yes	32 (22.4%)	93 (65%)	18 (12.6%)	0.94
No	4 (11.1%)	13 (12.3%)	3 (14.3%)	

Diabetes mellitus				
Yes	22 (26.5%)	53 (63.9%)	8 (9.6%)	0.23
No	14 (38.9%)	53 (50.5%)	13 (61.9%)	

BMI				
Obese	16 (37.2%)	22 (51.2%)	5 (11.6%)	0.02
Nonobese	20 (16.7%)	84 (70.0%)	16 (13.3%)	

Dialysis period	4.21 + 3.02	4.66 + 3.4	5 + 4.8	0.69

^*∗*^Kruskal–Wallis test; ^*∗∗*^chi-square test.

**Table 3:** Markers of mineral metabolism, liver function, renal function, age, and dialysis period according to serum 25 (OH)D levels.

	Severely deficient <10 ng/ml	Deficient 10–30 ng/ml	Normal >30 ng/ml	*P* values^*∗*^
	*n* = 36	*n* = 106	*n* = 21	
Calcium (mg/dL)	8.8 ± 0.9	9.5 ± 0.9	8.9 ± 0.8	0.250
Phosphorus (mg/dL)	4.6 ± 1.0	4.8 ± 1.3	4.5 ± 1.2	0.743
PTH (pg/ml)	498.9 ± 485.7	468.1 ± 628.4	426.3 ± 282.8	0.582
Alkaline phosphatase (U/L)	125.8 ± 104.4	140 ± 250.9	138.6 ± 118.8	0.236
Albumin (g/dL)	3.7 ± 0.33	3.8 ± 0.39	3.7 ± 0.5	0.094
AST (U/L)	12.2 ± 5.2	13.7 ± 5.9	15.1 ± 5.1	0.150
ALT (U/L)	10.9 ± 7.5	11.9 ± 7.1	12.9 ± 8.4	0.160
AST/ALT	1.3 ± 0.6	1.3 ± 0.5	1.4 ± 0.7	0.636

^*∗*^Kruskal–Wallis test;, PTH: parathyroid hormone; ALP: alkaline phosphatase; ALT: alanine transaminase; AST: aspartate transaminase.

**Table 4:** Spearman's correlation of 25 (OH)D levels and various clinical and biochemical parameters.

Parameter	Correlation coefficient	*P* value^*∗*^
Age (year)	−0.21	0.008
Dialysis period (year)	−0.016	0.836
Calcium (mg/dL)	0.031	0.836
Phosphorus (mg/dL)	0.020	0.799
PTH (pg/dL)	0.024	0.763
ALP (U/L)	0.05	0.530
Albumin (g/dL)	0.142	0.070
AST (U/L)	0.186	0.033
ALT (U/L)	0.199	0.011
AST/ALT	−0.023	0.777

^*∗*^Spearman's rank tests; PTH: parathyroid hormone; ALP: alkaline phosphatase; ALT: alanine transaminase; AST: aspartate transaminase.

**Table 5:** Multiple linear regression for predictors of 25 (OH)D level.

Constant	Beta	*R* square	*p* value
Age	−0.106	0.086	0.236
Gender	0.203		0.013
Diabetes mellitus	−0.122		0.143
Dialysis duration (years)	0.068		0.417
Calcium	−0.007		0.932
Phosphorous	0.045		0.603
ALP	0.075		0.415
Serum albumin	0.023		0.784
Serum PTH	−0.096		0.303

PTH: parathyroid hormone; ALP: alkaline phosphatase.

## Data Availability

The data used to support the findings of this study are available from the corresponding author upon request.
